# Radiation safety: knowledge, attitudes, practices and perceived socioeconomic impact in a limited-resource radiotherapy setting

**DOI:** 10.3332/ecancer.2025.1855

**Published:** 2025-02-20

**Authors:** Kofi Adesi Kyei, Hannah Boateng Addo, Joseph Daniels

**Affiliations:** 1 National Radiotherapy, Oncology and Nuclear Medicine Centre, Korle Bu Teaching Hospital, Box KB 369, Accra, Ghana; 2 Accra Business School, Leaders Factory, Spintex, PMB CT 170 Cantoment, Accra, Ghana; 3 Department of Radiography, University of Ghana, Box KB 143, Legon, Ghana; a https://orcid.org/0000-0003-3485-5368; b https://orcid.org/0000-0002-1466-150X

**Keywords:** radiation safety, radiation protection, radiotherapy, ‘health knowledge, attitudes and practices’, workplace safety, low-income populations, occupational health

## Abstract

Healthcare workers in teaching-hospital settings face numerous occupational hazards, necessitating comprehensive safety protocols to protect both staff and patients. Radiation safety is particularly critical in regions like sub-Saharan Africa, where the sharp rise in radiological procedures and radiation treatments demands stringent protocols to mitigate health risks. The study aimed to assess the knowledge, attitudes and practices related to radiation safety among healthcare workers in a limited-resource setting, as well as evaluate the perceived socioeconomic impact of implementing radiation safety protocols. The research was a quantitative case study of one of the largest radiotherapy centres in Africa. Participants were selected using a stratified random sampling technique. Data were collected using a modified structured questionnaire based on the validated International Atomic Energy Agency radiation safety knowledge, attitudes and practice questionnaire. Data were analysed with the Statistical Package for Social Sciences software. Both descriptive and inferential statistical analyses were performed. Data were summarized using frequencies, percentages, means and standard deviations. The study involved 78 participants, comprising 13 physicians, 40 nurses and 25 other health workers. In all, 53.8% were males, whereas 46.2% were females. The mean age was 24.9 years (SD 4.7) ranging from 23 to 47 years. A significant majority (82%) were knowledgeable about effective ways of reducing radiation exposure. All participants considered radiation safety extremely important, with 55% feeling extremely confident in their ability to practice radiation safety measures. The majority (92%) believed that radiation safety was a shared responsibility within the organization. Only 51% frequently checked radiation safety equipment. There was a strong consensus that these protocols positively affect healthcare worker well-being (a mean score of 4.49) and benefit patient care (a mean score of 4.45). Implementation of workplace safety protocols was perceived to improve worker well-being (B = 0.337 and *p* = 0.001) and benefit patient care (B = 0.391 and *p* = 0.014). The study highlights a high level of knowledge and positive attitudes towards radiation safety among healthcare workers in a limited-resource radiotherapy setting. While most participants recognized the importance of radiation safety and its shared responsibility, less than half regularly checked safety equipment. There were significant perceptions of the socioeconomic benefits of implementing safety protocols.

## Introduction

Healthcare workers in large-hospital settings are exposed to a myriad of occupational hazards, including infectious diseases, hazardous substances and potential radiation exposure, which collectively contribute to a challenging and high-risk work environment [1]. To protect the health and well-being of both patients and healthcare personnel, healthcare facilities implement comprehensive safety protocols aimed at maintaining a safe and healthy work environment [2]. Adherence to these protocols is crucial to prevent the spread of infectious diseases, reduce the incidence of workplace accidents and ensure overall safety [3, 4]. Such protocols are indispensable for enabling healthcare professionals to provide optimal patient care [5].

The utilization of personal protective equipment (PPE), including masks, gowns, gloves and other protective clothing, constitutes an essential safety routine to shield healthcare workers from hazardous substances [6, 7]. Additional safety practices in hospitals include preventing patient falls [8] and careful handling of hazardous materials [9]. These measures are not only essential for ensuring the safety of staff, patients and their relatives but also facilitate the delivery of high-quality patient care.

Radiation safety and occupational health protocols are fundamental in ensuring the well-being of healthcare workers, especially in regions with limited resources and high disease burdens. The application of these protocols in teaching hospitals is vital due to the dual role these institutions play in providing patient care and training healthcare professionals. In sub-Saharan Africa, the rapid expansion of medical facilities and the increasing use of diagnostic and therapeutic radiation services underscore the necessity for stringent radiation safety measures. The global burden of disease necessitates the use of advanced medical technologies, including radiological procedures and radiotherapy techniques, which inherently involve exposure to ionizing radiation. Approximately 3.6 billion diagnostic medical examinations involving ionizing radiation, such as X-rays, are performed annually worldwide, with a significant portion occurring in low- and middle-income countries [10]. Approximately 50%–60% of cancer patients require radiation therapy at some point during their treatment either with curative or palliative intent [11]. Despite the benefits of radiotherapy as a cornerstone of cancer management, inadequate safety measures can lead to significant health risks. Radiation safety is a critical component of healthcare delivery, particularly in radiotherapy settings where both patients and healthcare workers are exposed to ionizing radiation. Potential health risks associated with radiation exposure, such as cancer, genetic mutations, chronic dermatitis and infertility, necessitate robust safety protocols.

In the context of the National Radiotherapy, Oncology and Nuclear Medicine Centre (NRONMC) at the Korle-Bu Teaching Hospital (KBTH) in Ghana, radiation safety protocols are of paramount importance. Notably, NRONMC operates under a structured radiation safety program guided by national regulations and international standards. The facility employs a designated radiation safety officer who oversees compliance with safety protocols, conducts regular audits and ensures adherence to permissible radiation dose limits. Ghana’s radiation safety practices are regulated by the Ghana Atomic Energy Commission, which provides oversight and enforces compliance through licensing, inspections and training. This regulatory framework is further supported by guidelines from the International Atomic Energy Agency (IAEA). The radiation safety program at NRONMC emphasises core radiation protection principles, including time, distance, shielding and continuous monitoring to safeguard both healthcare workers and patients from excessive radiation exposure.

Undoubtedly, radiation therapy poses significant risks to both patients and medical professionals [12]. Effective radiation protection protocols, including the use of PPE, radiation monitoring, contamination control, radiation shielding, proper patient positioning, comprehensive training and emergency preparedness, are essential to minimize these risks and ensure safety [13–18]. Despite the critical importance of these protocols, their implementation can be challenging due to the need to balance safety with the provision of prompt, high-quality care [19]. In sub-Saharan African countries, the implementation of radiation safety measures faces unique challenges, including limited financial resources, inadequate infrastructure and insufficient training of healthcare personnel. Gaps in radiation safety knowledge, inconsistent safety practices and negative attitudes can impede the effective implementation of radiation safety protocols, potentially endangering patients and hospital staff.

The study aimed to assess the knowledge, attitudes and practices related to radiation safety among healthcare workers in a limited-resource radiotherapy setting. In addition, the study evaluated the perceived socioeconomic impact of implementing radiation safety protocols. The study provides insights into areas that require intervention, ensuring a safer environment for both healthcare workers and patients. The case study is vital in highlighting the gaps in radiation safety at low-income radiotherapy centres, where resource limitations present unique challenges. By evaluating healthcare workers’ perceptions of the socioeconomic impact of implementing safety protocols, the study seeks to inform policy changes that prioritize radiation safety while considering economic realities.

## Methods

### Study design and setting

This research was a quantitative case study conducted at KBTH in Ghana. This hospital is the largest tertiary healthcare facility in the country, one of the premier healthcare institutions in West Africa and the third-largest hospital in Africa. The hospital has several centres of excellence, one of which is NRONMC, established in 1997. The centre manages cancer patients with tumours of all sites, employing modalities such as chemotherapy, immunotherapy, targeted and hormonal therapy, brachytherapy and external beam radiotherapy. At NRONMC, new employees undergo a structured radiation safety on-boarding program led by the radiation safety officer. This program includes theoretical and practical training on radiation protection principles, emergency procedures, proper use of PPE and equipment monitoring protocols. In addition, all staff are required to participate in annual refresher courses designed to reinforce safety practices and update them on any changes to local or international guidelines. These training sessions are complemented by periodic drills to ensure preparedness for radiation-related emergencies. The comprehensive training framework at NRONMC is designed to ensure that all employees have the necessary knowledge and skills to maintain high standards of radiation safety in their roles.

### Study population

The study population comprised permanent medical staff of the radiotherapy centre at KBTH stratified into three groups: physicians, nurses and other healthcare workers with at least 1 year of work experience at KBTH. The latter criterion ensured that participants had sufficient exposure to the work environment and relevant radiation safety protocols. Exclusion criteria included temporary or contractual staff, nonclinical staff (e.g., janitorial staff or external contractors), employees with less than 1 year of experience, individuals who did not provide written informed consent and employees who were on extended leave during the study period.

### Study size

Stratified random sampling was used to select the study participants. The study population was stratified into three groups (strata) according to job category, namely, physicians, nurses and other healthcare workers. Study participants were then randomly selected from each group. This sampling approach was adopted to account for the heterogeneous nature of the study population, ensuring the adequate representation of natural subgroups in the study population. The total eligible workforce at the study site was 110 employees comprising 18 physicians, 55 nurses and 37 other (healthcare) workers. Based on Yamane’s formula [20], the appropriate study size was determined to be 87 participants, stratified into 14 physicians, 44 nurses and 29 other (healthcare) workers.

### Data collection

Data were collected using a structured questionnaire based on the validated IAEA radiation safety knowledge, attitudes and practice questionnaire to assess information from respondents regarding radiation safety at the study site. Questionnaires were administered to 17 physicians, 45 nurses and 33 other healthcare workers either in-person or electronically based on each participant’s preference. The questionnaire was specifically designed to assess various aspects of radiation safety knowledge, attitudes, practices and the perceived socioeconomic impact of implementing safety protocols among healthcare workers at KBTH. The first section gathered demographic data, including sex, age, marital status and educational level, whereas the second section evaluated respondents’ knowledge of radiation safety, focusing on methods to reduce exposure, types of harmful radiation, permissible dose limits and key principles of radiation protection. The third section measured attitudes toward radiation safety, including its importance, confidence in practicing safety policies, willingness to report incidents, frequency of knowledge updates and views on shared responsibility. The fourth section examined radiation safety practices, such as the frequency of equipment checks, understanding of dose limits, use of personal protective equipment and knowledge of emergency procedures. The final section assessed the perceived socioeconomic impact of safety protocols on healthcare worker well-being, patient care and the justification of associated costs.

### Data analysis

Data cleaning was conducted to identify and correct errors, inconsistencies and outliers. The Statistical Package for Social Sciences software was used to analyse data. The pattern of responses to each questionnaire item was described using frequency distributions. Demographic data (age, gender and marital status) were summarized using frequencies, percentages, means and standard deviations. Multiple regression analysis was used to examine the relationship between different aspects of safety protocols and the socioeconomic impact of their implementation. Simple imputation methods such as mean substitution were used in addressing the challenge of missing data.

### Bias

Selection bias was minimized by using a stratified random sampling technique to ensure a representative sample of eligible healthcare workers at the study site. Response bias was reduced by ensuring the anonymity and confidentiality of completed questionnaires to encourage honest responses. The use of the validated, reliable IAEA KAP questionnaire ensured accurate measurement of the variables of interest.

### Ethical considerations

Ethical approval was obtained from the Institutional Review Board of the Accra Business School prior to the commencement of the study. All participants provided written informed consent. The privacy of participants and the confidentiality of their data were maintained by anonymizing responses and storing data securely.

## Results

### Baseline characteristics of the study population

Questionnaires were administered to 95 participants at an 82% response rate. The study involved 13 physicians, 40 nurses and 25 other health workers (*N* = 78). Table 1 summarizes the demographic and professional characteristics of the participants. Overall, there were 53.8% males and 46.2% females with a mean age of 24.9 years (SD 4.7) ranging from 23 to 47 years. Only 17 (21.8%) were married. In all, 58 participants (74.3%) held bachelor’s degrees, whereas 18 (23.1%) had master’s degrees. Also, 65 (83.3%) had ≤5 years’ working experience, whereas 6 (7.7%) had over 10 years of experience.

### Descriptive analysis of radiation safety knowledge

Table 2 presents the scores of participants on their radiation safety knowledge, highlighting the frequency and percentage distribution of responses to five key questions. A significant majority (82%) correctly identified that ‘all the above’ (limiting time of exposure, increasing distance and using PPE) were effective ways to reduce radiation exposure. A smaller portion chose ‘limiting the time of exposure’ (12%) and ‘increasing the distance from the source of radiation’ (6%), whereas none (0%) selected ‘using personal protective equipment (PPE)’ alone. Regarding the most ionizing type of radiation with the greatest potential for harm, 37% correctly selected ‘alpha particles’. Meanwhile, 63% incorrectly selected ‘gamma rays’, and no participants chose ‘beta particles’ or ‘X-rays’. This indicates a significant misconception among most respondents, who mistakenly believed that gamma rays were more ionizing than alpha particles. Participants’ knowledge of the regulatory limits for occupational radiation exposure was also tested. In all, 54% (*n* = 42) correctly answered that the maximum permissible annual dose limit for occupational exposure was 50 mSv, reflecting familiarity and reasonable awareness of safety regulations and dose limits. Regarding the recommended dose limit for a pregnant radiation worker during the entire gestational period, only 19% correctly identified ‘5 mSv’. The majority (51%) incorrectly chose ‘1 mSv’, followed by ‘0.1 mSv’ (29%). This indicates a widespread misconception among the participants regarding the correct dose limit for pregnant radiation workers. All participants (100%) correctly identified ‘concentration’ as not being a principle of radiation protection, demonstrating a comprehensive understanding of the fundamental principles of radiation protection among all respondents.

### Descriptive analysis of radiation safety attitudes

Table 3 illustrates participants’ perceptions and attitudes towards radiation safety. All participants considered radiation safety to be ‘extremely important’, indicating unanimous recognition of its critical significance. Regarding confidence in practicing radiation safety, 55% of the participants felt ‘extremely confident’, whereas 36% were ‘very confident’, suggesting a high overall confidence level. However, 9% were ‘not confident at all’, highlighting a minority with significant lack of confidence in practicing radiation safety. Regarding the reporting of incidents or near-misses, 71% were proactive, indicating that they ‘actively encourage others to report incidents as well’. Also, 15% indicated that they ‘would report only if required’. No participants ‘preferred not reporting’, demonstrating a strong culture of safety and accountability. In terms of knowledge updates, 42% reviewed their radiation safety knowledge ‘frequently’, whereas 49% did so ‘occasionally’. A small fraction (9%) reviewed their knowledge ‘rarely’, suggesting the need for more regular knowledge updates among some staff. A vast majority (92%) believed that radiation safety was a ‘shared responsibility for all individuals in the organization, including management’, whereas 8% thought it was a shared duty among those who work with radiation. No one felt that it was solely the responsibility of the radiation safety officer, indicating a widespread understanding of collective responsibility for radiation safety.

### Descriptive analysis of radiation safety practices

Table 4 provides insights into the frequency of radiation safety practices among participants. The majority (51%) reported checking equipment ‘frequently’, indicating a good practice of regular monitoring. However, 37% stated that they ‘rarely’ check the equipment, and 12% do so ‘occasionally’, suggesting a need for improved consistency in equipment checks. Pertaining to knowledge of radiation dose limits for occupational exposure, 56% expressed confidence in their ability to determine these limits. Meanwhile, 31% had a basic understanding but lacked confidence, and 13% had no idea about these dose limits. None of the participants had received specialized training in this area, indicating a gap in advanced education on radiation dose limits. Regarding the use of PPE when working with radiation, 56% reported wearing PPE ‘frequently’, demonstrating good compliance with safety protocols. Conversely, 23% wore PPE ‘occasionally’, 12% ‘rarely’ and 9% ‘never’, highlighting the need for more consistent use of PPE among some participants. In all, 37% reported reviewing and updating their knowledge of radiation safety practices ‘frequently’, whereas 40% did so ‘occasionally’ and 23% ‘rarely’. No participants reported ‘Never’ reviewing or updating their knowledge, but the varying frequencies suggest room for improvement in maintaining up-to-date knowledge. Regarding knowledge of procedures for responding to a radiation emergency, 38% were confident in their ability to respond, and an additional 6% had received specialized training. A significant proportion (49%) had a basic understanding but lacked confidence. Another 6% had no idea about emergency response procedures, indicating a need for enhanced training and preparedness for radiation emergencies.

### Perceived socioeconomic impact of implementing work and safety protocols

Table 5 summarizes participants’ perceptions of the socioeconomic impact of implementing work and safety protocols in the hospital. A significant majority (84%) believed that work and safety protocols effectively reduced workplace injuries and accidents, with 56% ‘Strongly agreeing’ and 28% ‘Agreeing’. Only 9% ‘Strongly disagreed’ and 6% were ‘Neutral’, mean score of 4.23 (SD 1.2). The implementation of safety protocols was seen as having a positive impact on the well-being of healthcare workers, mean score of 4.49 (SD 0.5). Most participants (91%) believed that the implementation of safety protocols was beneficial to patient care, with 54% ‘Strongly agreeing’ and 37% ‘Agreeing’. A small percentage (9%) were ‘Neutral’, resulting in a mean score of 4.45 (SD 0.7). The cost of implementing work and safety protocols was perceived to be justified by the benefits it provides, mean score of 4.62 (SD 0.5). Most participants (62%) ‘Strongly agreed’ and 38% ‘Agreed’. Participants widely recognized the importance of regularly evaluating the socioeconomic impact of workplace injuries and accidents, mean score of 4.46 (SD 0.5).

### Multiple regression analysis of perceived socioeconomic impact of implementation of safety protocols

The predictor ‘work and safety protocols: reduce injuries’ had a negative unstandardized coefficient (−0.174), which was statistically significant (*p* = 0.039) (Table 6). ‘Work and safety protocols: affect worker well-being’ had a positive unstandardized coefficient (0.337) and was also significant (*p* = 0.001), suggesting that improving worker well-being was perceived to increase the socioeconomic impact of implementing safety protocols. Similarly, ‘work and safety protocols: benefit patient care’ showed a positive and significant relationship (*b* = 0.391 and *p* = 0.014), indicating that enhancing patient care through safety protocols was also perceived to increase the socioeconomic impact of workplace safety protocols. Finally, the ‘cost of implementing protocols: justified by benefits’ had a positive unstandardized coefficient (0.245) and was marginally significant (*p* = 0.053), suggesting that while the costs are justified by the benefits, they still impact socioeconomic factors.

## Discussion

### Key findings

The study assessed the knowledge, attitudes and practices related to radiation safety among healthcare workers at a major radiotherapy centre in Africa. The study included 78 participants, comprising 13 physicians, 40 nurses and 25 other health workers. In all, 53.8% were males, whereas 46.2% were females. The mean age was 24.9 years (SD 4.7) ranging from 23 to 47 years. Most held bachelor’s degrees (74.3%) and had ≤5 years of working experience (83.3%). A significant majority (82%) were knowledgeable about effective ways of reducing radiation exposure. All participants considered radiation safety extremely important, with 55% feeling extremely confident in their ability to practice radiation safety measures. The majority, actively encouraged reporting incidents or near-misses (71%) and believed that radiation safety was a shared responsibility within the organization (92%). However, only 51% frequently checked radiation safety equipment. Knowledge of correct procedures for radiation emergencies varied, with 38% being confident and 49% having a basic understanding but lacking confidence. Most of the participants (84%) believed that work and safety protocols effectively reduced workplace injuries. There was a strong consensus that these protocols positively affect healthcare worker well-being (a mean score of 4.49) and benefit patient care (a mean score of 4.45). Regression analysis showed that the implementation of workplace safety protocols was perceived to improve worker well-being (B = 0.337 and *p* = 0.001) and benefit patient care (B = 0.391 and *p* = 0.014). The cost of implementing protocols, while justified by the benefits, had a marginally significant positive impact (B = 0.245 and *p* = 0.053).

### Radiation safety knowledge

In all, 82% correctly identified multiple strategies to reduce radiation exposure, namely, limiting time, increasing distance and using PPE. This reflects knowledge of the core principles of radiation safety – time, distance and shielding – which are internationally recognized for minimizing radiation exposure risks [21]. However, it is concerning that 12% only selected limiting the time of exposure as effective, while 6% chose increasing the distance alone, indicating a partial understanding among a small segment of the workforce. This suggests that while the majority have grasped the comprehensive approach to radiation safety, a minority may still benefit from further education or reinforcement on the importance of integrating all these methods. A more concerning gap in knowledge was found in participants' understanding of which type of radiation is the most ionizing. Only 37% correctly identified alpha particles, with 63% incorrectly choosing gamma rays. Alpha particles are indeed the most ionizing, though they have low penetration power, and understanding the distinction between ionizing potential and penetration is crucial for professionals in radiation-heavy environments [22]. The misconception regarding gamma rays, which have high penetration power but are less ionizing than alpha particles, points to a potential misunderstanding of radiation properties that could have safety implications in practice. This gap mirrors findings from similar studies, where healthcare workers sometimes misunderstand key concepts about the nature of radiation and its biological effects [23].

The study also assessed participants’ knowledge of maximum permissible annual radiation doses for occupational exposure, with 54% correctly identifying the limit of 50 mSv. This indicates a reasonable level of awareness of safety regulations among participants, which is a positive finding. Familiarity with dose limits is a critical component of radiation safety since exceeding these thresholds can lead to significant health risks, including cancer and other radiation-induced injuries [24]. However, the finding that nearly half of the participants were either unaware of or confused about these limits suggests that continued professional education and frequent reinforcement of regulatory guidelines may be necessary. One of the most striking gaps in knowledge was related to dose limits for pregnant radiation workers. Only 19% knew the correct dose limit of 5 mSv for the entire gestational period, while the majority (51%) incorrectly selected 1 mSv, and a considerable portion (29%) chose 0.1 mSv. This is a critical gap, as pregnancy is a particularly sensitive period in which radiation exposure must be minimized to protect both the mother and the developing foetus from potential harm [25]. The widespread misconception about these limits could lead to either unnecessary restrictions on pregnant workers or inadequate protection, both of which could have negative consequences for health and workforce management.

All participants correctly identified ‘concentration’ as not being a principle of radiation protection. This also reflects a comprehensive understanding of the three main principles of radiation protection – time, distance and shielding [26]. This demonstrates that at a fundamental level, healthcare workers in this setting are well-versed in the basic concepts necessary for minimizing radiation exposure, which is crucial for both their safety and that of their patients. These results underscore both the strengths and challenges in radiation safety knowledge among healthcare workers in limited-resource settings. The correct identification of radiation safety methods by most participants is encouraging, as it reflects a strong foundation in safety practices. The discrepancies in understanding of dose limits may reflect a broader issue of access to up-to-date safety guidelines in limited-resource settings. In regions where educational resources, training opportunities and regulatory enforcement may be lacking, healthcare workers may not have regular access to continuing education on radiation safety practices [27]. This raises the need for targeted interventions, including workshops, safety training programs and the dissemination of clear, up-to-date safety guidelines to bridge these gaps.

### Radiation safety attitudes

Participants demonstrated a high level of awareness regarding the importance of radiation safety, with 100% considering it extremely important. Confidence in practicing radiation safety was also high, with 55% feeling extremely confident and 36% very confident. However, 9% lacked confidence, indicating the need for targeted interventions to boost confidence levels. The willingness to report incidents or near-misses was high, with 71% actively encouraging others to report. This positive safety culture is essential for effective radiation protection. The unanimous recognition of radiation safety as ‘extremely important’ underscores a strong awareness of its critical significance in the radiotherapy setting. This aligns with findings from similar studies in both high- and low-income settings, where there is a broad consensus on the importance of radiation safety [28, 29]. For instance, a study in a high-income setting found that all surveyed healthcare workers recognized radiation safety as crucial, reflecting a universal understanding across different economic contexts [30]. In all, 55% felt ‘extremely confident’, and 36% were ‘very confident’ in their ability to practice radiation safety. This high level of confidence is promising, indicating that most healthcare workers are well-equipped to adhere to safety protocols. However, 9% who were ‘not confident at all’ highlight a critical gap that requires attention. Research in similar settings has shown that confidence levels in safety practices can significantly impact compliance and overall safety [31]. In contrast, a study from a high-income country revealed a slightly higher confidence level among healthcare workers, suggesting that resource availability and training might influence confidence [32]. In high-income settings, where resources are more abundant, confidence in safety practices tends to be higher, partly due to more frequent and comprehensive training [33]. Conversely, low-income settings may face challenges such as inadequate training resources and lower confidence levels, necessitating focused capacity-building efforts.

The study also showed that 71% of the participants actively encouraged others to report incidents or near-misses, while 14% believed that it was important to report, and 15% would report only if required. This high rate of proactive reporting is indicative of a robust safety culture. Studies have demonstrated that a culture of transparency and reporting is essential for improving safety outcomes [34]. However, 15% who report only if required could suggest an area where further emphasis on the importance of reporting might be needed. High-income settings often benefit from established incident reporting systems and a strong culture of safety [35]. In contrast, limited-resource settings may struggle with fewer formal reporting mechanisms. The strong reporting culture found here is indicative of a positive safety climate, but the study’s findings suggest that reinforcing the importance of reporting, especially among those who report only if required, could further enhance safety practices. Regarding the frequency of knowledge updates, 42% reviewed their radiation safety knowledge ‘frequently’, and 49% did so ‘occasionally’, while 9% reviewed their knowledge ‘rarely’. The need for more regular updates is evident, as continual education is crucial for maintaining high standards of safety [36]. Similar studies have found that regular updates in safety knowledge are correlated with better adherence to protocols [37]. The frequency of knowledge updates in this study highlights a need for more regular training. In high-income settings, ongoing professional development is often more systematic and frequent [38]. Limited-resource settings can benefit from integrating more structured and regular training opportunities to bridge this gap. A vast majority (92%) believed that radiation safety was a ‘shared responsibility for all individuals in the organization, including management’, while 8% thought that it was shared only among those who work with radiation. This widespread understanding of collective responsibility is consistent with best practices in radiation safety [39]. The concept of shared responsibility for radiation safety was well-understood among participants, reflecting a critical component of effective safety protocols. High-income settings often have more comprehensive policies and management systems in place to support this understanding, while low-income settings might need additional support to fully implement these concepts [40]. Comparatively, high-income settings also emphasise shared responsibility, but there is often more infrastructure to support this understanding through formal policies and training [41].

### Radiation safety practices

Most participants (51%) reported checking radiation safety equipment ‘frequently’, which is indicative of a generally good practice of regular monitoring. However, a combined 49% reported either ‘rarely’ or ‘occasionally’ checking equipment, revealing a potential area for improvement. This inconsistency in equipment check points to the need for regular and mandatory safety checks. Regular equipment checks are crucial for ensuring safety and preventing radiation-related incidents [42, 43]. Studies from high-income settings generally show a higher frequency of equipment checks due to better resource availability and more rigorous safety protocols [44, 45]. In contrast, low-resource settings often face challenges such as equipment malfunctions and insufficient monitoring practices, which can impact safety [46, 47]. The compliance with wearing PPE was generally good, with 56% of participants wearing PPE ‘frequently’. Regular and proper use of PPE is essential for protecting healthcare workers from radiation exposure [34]. High-income settings often report higher compliance with PPE use due to better enforcement of safety protocols and availability of equipment [37]. Low-resource settings may face difficulties in maintaining consistent PPE use due to shortages and less rigorous safety practices [48].

Regular review and update of radiation safety knowledge were also found to be variable, with 37% doing so frequently, but 23% rarely updating their knowledge. While no participants reported ‘never’ updating their knowledge, the varying frequencies indicate that not all staff maintain up-to-date knowledge consistently. Regular updates are vital for staying informed about the latest safety protocols and advancements [38]. High-resource settings often have formal continuing education programs that ensure frequent updates, whereas low-resource settings may lack such structured opportunities [49, 50]. Ensuring continuous education and training is crucial for maintaining high safety standards.

The availability of structured on-boarding and recurring training programs at NRONMC reflects a commitment to fostering a robust safety culture. However, the variability in confidence levels and adherence to certain safety practices, such as equipment checks, and PPE use is worrisome. Periodic evaluation of radiation safety training programs is required, to ensure that they effectively address knowledge gaps and translate into consistent safety practices. Enhancing the content of refresher courses and incorporating advanced simulation-based methods may further improve preparedness and compliance. In addition, a more frequent and personalized approach to training could be beneficial in addressing specific challenges faced by individual staff members.

### Perceived socioeconomic impact of implementing work and safety protocols

Most participants (84%) believed that work and safety protocols were effective in reducing workplace injuries and accidents. This reflects a strong perception of protocol effectiveness in improving safety. The high mean score (4.23) indicates that participants largely view these protocols as beneficial for injury reduction. In high-income settings, similar studies have shown that well-implemented safety protocols significantly decrease workplace accidents due to rigorous enforcement and regular updates [51, 52]. Conversely, low-resource settings may struggle with implementing and maintaining such protocols effectively due to limited resources and training [53, 54]. The positive perception in this study aligns with findings from other low-income settings where improvements in safety protocols are associated with reduced injuries, although challenges persist [55]. The implementation of safety protocols is perceived to have a positive effect on the well-being of healthcare workers, with a mean score of 4.49, reflecting strong agreement. This suggests that participants believed these protocols contribute to a better work environment and reduced stress among workers. In high-income settings, safety protocols are often associated with improved worker satisfaction and reduced burnout [56, 57]. The positive impact observed in this study is consistent with findings from other low-income settings where the introduction of safety measures has been linked to better job satisfaction and reduced psychological stress [58, 59]. However, disparities in resources and support can affect the extent of these benefits in low-resource settings [60].

The majority (91%) of participants perceived that safety protocols positively impact patient care, with a mean score of 4.45. This indicates a strong consensus that the implementation of safety measures enhances the quality of care provided to patients. High-income settings have shown that effective safety protocols contribute to improved patient outcomes due to better overall safety standards and practices [61, 62]. In low-income settings, while the positive impact on patient care is similarly recognized, challenges, such as resource limitations and less rigorous implementation, may affect the degree of this benefit [38, 63]. The study’s results align with other research suggesting that even in resource-limited environments, safety protocols can still positively affect patient care [64]. Participants generally perceived the costs of implementing safety protocols as justified by the benefits, with a high mean score of 4.62. This suggests strong agreement that the investment in safety measures is worthwhile. In high-income settings, the justification of costs is often supported by comprehensive cost–benefit analyses showing significant returns in terms of reduced accidents and improved care [65, 66]. In low-income settings, while the justification of costs is recognized, financial constraints may make it challenging to fully implement and maintain these protocols [67, 68]. The study’s findings reflect a broader consensus that the benefits of safety protocols outweigh their costs, even in resource-limited environments. High-income settings often have established processes for regular evaluation and adjustment of safety protocols [69]. In low-resource settings, while there is a recognition of the need for regular evaluation, practical challenges, such as limited resources and infrastructure, may impact the frequency and effectiveness of these evaluations [70, 71]. The strong agreement in this study underscores the importance of continuous assessment, which is crucial for maintaining and improving safety standards. There was a strong perception that improving worker well-being through safety protocols enhances the overall socioeconomic impact. This aligns with research showing that better worker well-being is associated with increased job satisfaction and productivity [72, 73]. Enhancing patient care through safety protocols was perceived to have a significant positive impact. This reflects findings that effective safety measures contribute to better patient outcomes [74, 75]. Regarding the cost of implementing safety protocols. The marginally significant positive coefficient indicates that while the costs are generally perceived as justified, the impact on socioeconomic factors may still be subject to variability based on financial constraints and resource availability.

## Limitations

The relatively small sample size may affect statistical power and broader applicability. Potential confounding variables, such as differences in job experience and training, may not have been fully accounted for. The cross-sectional design limits the ability to establish cause-and-effect relationships and capture changes over time. The reliance on self-reported data may introduce biases such as social desirability bias, where participants might respond in a manner they perceive as favourable or expected.

## Recommendations

The implementation of regular and mandatory training sessions is recommended to address knowledge gaps and ensure that all staff are up-to-date with the latest safety protocols. Establishing a systematic schedule for radiation safety equipment checks will ensure consistency and compliance, while strengthening the enforcement of PPE protocols will ensure that all staff consistently use protective equipment. In addition, encouraging continuous education and regular updates of radiation safety knowledge among all healthcare workers is essential. Fostering a comprehensive safety culture that encourages the reporting of incidents and near-misses will ensure a proactive approach to radiation safety. Collaboration among healthcare workers, management and stakeholders through regular meetings and open communication will aid in resolving issues and updating safety measures. Finally, continuous investigation and evaluation of work and safety protocols will identify problem areas and guide future interventions. Future research should include a larger and more diverse sample to enhance the generalizability of the findings. This could involve multiple healthcare facilities to capture a broader range of perceptions and experiences. Subsequent studies should incorporate qualitative methods, such as interviews or focus groups, to gain a deeper understanding of the factors influencing perceptions of safety protocols and to explore potential barriers to effective implementation.

## Conclusion

This study underscores the high level of knowledge and positive attitudes toward radiation safety among healthcare workers in a resource-limited radiotherapy setting. However, specific knowledge gaps and inconsistencies in safety practices remain, necessitating targeted interventions and a culture of continuous education. Although most participants recognized the shared responsibility of radiation safety, less than half consistently performed equipment checks and compliance with PPE usage varied. The findings emphasise the importance of mandatory, ongoing training to keep staff informed about updated safety protocols and ensure consistent adherence to best practices. In addition, the study reveals that the perceived socioeconomic benefits of implementing safety protocols – despite the associated costs – are significant, positively impacting both healthcare worker well-being and patient care.

To further enhance safety standards, systematic equipment checks and strict enforcement of PPE usage must be prioritized. Ultimately, this research highlights the critical role of robust safety protocols in safeguarding healthcare workers and delivering optimal patient outcomes in a teaching-hospital setting.

## Conflicts of interest

The authors declare no competing interest.

## Funding

This study did not receive any specific funding support from funding agencies in the public, commercial, or not-for-profit sectors.

## Data availability

The data used to support the findings of this study are available from the corresponding author upon reasonable request.

## Author contributions

KAK: Supervision, methodology, data analysis and writing – review and editing.

HBA: Conceptualization, data collection and writing – original draft.

JD: Conceptualization, methodology, data analysis and writing – original draft and review and editing.

## Figures and Tables

**Table 1.** Baseline characteristics of the study population (*N* = 78).

Characteristics	Frequency	Percentage (%)
Sex		
Male	42	53.8
Female	36	46.2
Age (years)		
20–25	56	71.8
26–30	16	20.5
36–40	4	5.1
>40 years	2	2.6
Marital status		
Married	17	21.8
Not married	61	78.2
Highest level of formal education		
Bachelor’s degree	58	74.3
Master’s degree	18	23.1
Doctorate degree	2	2.6
Years of experience		
1–5	65	83.3
6–10	7	9.0
>10	6	7.7

**Table 2.** Radiation safety knowledge among participants (N = 78).

Questionnaire items	Frequency	Percentage (%)
1. What is the most effective way to reduce exposure to radiation?		
A. Limiting the time of exposure	9	12
B. Increasing the distance from the source of radiation	5	6
C. Using personal protective equipment (PPE)	0	0
D. All the above	64	82
2. Which of the following types of radiation is the most ionizing and has the greatest potential for harm?		
A. Alpha particles	29	37
B. Beta particles	0	0
C. Gamma rays	49	63
D. X-rays	0	0
3. What is the maximum permissible annual dose limit for occupational exposure to radiation in the United States?		
A. 1 millisievert (mSv)	16	21
B. 5 mSv	20	26
C. 50 mSv	42	54
D. 100 mSv	0	0
4. What is the recommended dose limit for a pregnant radiation worker during the entire gestational period?		
A. 0.1 mSv	23	29
B. 1 mSv	40	51
C. 5 mSv	15	19
D. 50 mSv	0	0
5. Which of the following is not a principle of radiation protection?		
A. Time	0	0
B. Distance	0	0
C. Shielding	0	0
D. Concentration	78	100

The correct answers are highlighted

**Table 3.** Radiation safety attitude and perceptions (*N* = 78).

Questionnaire items	Frequency	Percentage (%)
6. How important do you think radiation safety is?		
A. Not important at all	0	0
B. Somewhat important	0	0
C. Very important	0	0
D. Extremely important	78	100
7. How confident are you in your ability to practice radiation safety?		
A. Not confident at all	7	9
B. Somewhat confident	0	0
C. Very confident	28	36
D. Extremely confident	43	55
8. Are you willing to report incidents or near-misses related to radiation safety?		
A. No, I prefer not to report	0	0
B. Yes, but only if it is required	12	15
C. Yes, I feel it is important to report	11	14
D. Yes, and I actively encourage others to report as well	55	71
9. How often do you review and update your knowledge of radiation safety?		
A. Never	0	0
B. Rarely	7	9
C. Occasionally	38	49
D. Frequently	33	42
E. No, it is solely the responsibility of the radiation safety officer	0	0
10. Do you think that radiation safety is a shared responsibility?		
A. Yes, but only in certain situations	0	0
B. Yes, it is a shared responsibility of everyone who works with radiation	6	8
C. Yes, and it is a shared responsibility for all individuals in the organization, including management	72	92

**Table 4.** Radiation safety practices (*N* = 78).

Questionnaire items	Frequency	Percentage (%)
11. How often do you check radiation safety equipment, such as radiation monitors?		
A. Never	0	0
B. Rarely	29	37
C. Occasionally	9	12
D. Frequently	40	51
12. Do you know the radiation dose limits for occupational exposure?		
A. No, I have no idea	10	13
B. I have a basic understanding, but not confident in my ability	24	31
C. Yes, I am confident in my ability to determine radiation dose limits	44	56
D. Yes, and I have received specialized training in this area	0	0
13. How often do you wear personal protective equipment when working with radiation?		
A. Never	**7**	9
B. Rarely	9	12
C. Occasionally	18	23
D. Frequently	44	56
14. How often do you review and update your knowledge of radiation safety practices?		
A. Never	0	0
B. Rarely	18	23
C. Occasionally	31	40
D. Frequently	29	37
15. Do you know the correct procedures for responding to a radiation emergency?		
A. No, I have no idea	5	6
B. I have a basic understanding, but not confident in my ability	38	49
C. Yes, I am confident in my ability to respond to a radiation emergency	30	38
D. Yes, and I have received specialized training in this area	5	6

**Table 5.** Perceived socioeconomic impact of implementation of work and safety protocols.

Questionnaire items		*N*	%	Mean	SD
16. The work and safety protocols in the hospital effectively reduce workplace injuries and accidents.	A. Strongly disagree	9	7	4.23	1.2
	B. Disagree	0	0		
	C. Neutral	6	5		
	D. Agree	28	22		
	E. Strongly agree	56	44		
17. The implementation of work and safety protocols in the hospital positively affects the well-being of healthcare workers.	A. Strongly disagree	0	0	4.49	0.5
	B. Disagree	0	0		
	C. Neutral	0	0		
	D. Agree	51	40		
	E. Strongly agree	49	38		
18. The implementation of work and safety protocols in the hospital is beneficial to patient care.	A. Strongly disagree	0	0	4.45	0.7
	B. Disagree	0	0		
	C. Neutral	9	7		
	D. Agree	37	29		
	E. Strongly agree	54	42		
19. The cost of implementing work and safety protocols in the hospital is justified by the benefits it provides.	A. Strongly disagree	0	0	4.62	0.5
	B. Disagree	0	0		
	C. Neutral	0	0		
	D. Agree	38	30		
	E. Strongly agree	62	48		
20. The socioeconomic impact of workplace injuries and accidents on healthcare workers and patient care is an important factor that should be evaluated regularly.	A. Strongly disagree	0	0	4.46	0.5
	B. Disagree	0	0		
	C. Neutral	0	0		
	D. Agree	54	42		
	E. Strongly agree	46	36		

**Table 6.** Regression analysis of the perceived socioeconomic impact of safety protocols on healthcare workers and patient care.

Predictor	Unstandardized coefficients (B)	Standardized coefficients (Beta)	*t*-value	*p*-value
(Constant)	0.811	0.681	1.191	0.238
Work and safety protocols: reduce injuries	−0.174	−0.410	−2.099	0.039
Work and safety protocols: affect worker well-being	0.337	0.338	3.420	0.001
Work and safety protocols: benefit patient care	0.391	0.513	2.506	0.014
Cost of implementing protocols: justified by benefits	0.245	0.239	1.968	0.053

Dependent variable: ‘The socioeconomic impact of workplace injuries and accidents on healthcare workers and patient care is an important factor that should be evaluated regularly’.
